# Inhibition of vascular smooth muscle growth via signaling crosstalk between AMP-activated protein kinase and cAMP-dependent protein kinase

**DOI:** 10.3389/fphys.2012.00409

**Published:** 2012-10-29

**Authors:** Joshua D. Stone, Avinash Narine, David A. Tulis

**Affiliations:** Department of Physiology, Brody School of Medicine, East Carolina UniversityGreenville, NC, USA

**Keywords:** AMPK, protein kinase A, protein phosphatase, proliferation, migration

## Abstract

Abnormal vascular smooth muscle (VSM) growth is central in the pathophysiology of vascular disease yet fully effective therapies to curb this growth are lacking. Recent findings from our lab and others support growth control of VSM by adenosine monophosphate (AMP)-based approaches including the metabolic sensor AMP-activated protein kinase (AMPK) and cAMP-dependent protein kinase (PKA). Molecular crosstalk between AMPK and PKA has been previously suggested, yet the extent to which this occurs and its biological significance in VSM remain unclear. Considering their common AMP backbone and similar signaling characteristics, we hypothesized that crosstalk exists between AMPK and PKA in the regulation of VSM growth. Using rat primary VSM cells (VSMC), the AMPK agonist AICAR increased AMPK activity and phosphorylation of the catalytic Thr172 site on AMPK. Interestingly, AICAR also phosphorylated a suspected PKA-inhibitory Ser485 site on AMPK, and these cumulative events were reversed by the PKA inhibitor PKI suggesting possible PKA-mediated regulation of AMPK. AICAR also increased PKA activity in a reversible fashion. The cAMP stimulator forskolin increased PKA activity and completely ameliorated Ser/Thr protein phosphatase-2C activity, suggesting a potential mechanism of AMPK modulation by PKA since inhibition of PKA by PKI reduced AMPK activity. Functionally, AMPK inhibited serum-stimulated cell cycle progression and cellular proliferation; however, PKA failed to do so. Moreover, AMPK and PKA reduced PDGF-β-stimulated VSMC migration. Collectively, these results show that AMPK is capable of reducing VSM growth in both anti-proliferative and anti-migratory fashion. Furthermore, these data suggest that AMPK may be modulated by PKA and that positive feedback may exist between these two systems. These findings reveal a discrete nexus between AMPK and PKA in VSM and provide basis for metabolically-directed targets in reducing pathologic VSM growth.

## Introduction

Aberrant vascular smooth muscle (VSM) growth is a pivotal mechanism underlying numerous pathologies including the evolution of atherosclerotic plaque, pressure- and/or flow-mediated hypertrophy, stent-induced hyperplasia, and iatrogenic restenosis after intervention (Majesky, 1994; Insull, 2009; Wang et al., 2010). The “de-differentiation” of VSM cells (VSMCs) from a normally cytostatic, contractile phenotype to a synthetic phenotype with enhanced proliferation, migration, and secretion of extracellular matrix is a major player in the establishment and progression of these disorders. In spite of many studies aimed at reducing the individual components that make up this activated phenotype, abnormal VSMC growth remains a critical contributor to vascular pathology and comprehensive, clinically feasible therapies have yet to be identified.

Adenosine monophosphate-activated protein kinase (AMPK) is a key metabolic sensor that responds to cellular energy depletion and stress by inhibiting many ATP-reducing processes, thereby promoting ATP synthetic processes within the cell (Rubin et al., 2005; Sanders et al., 2007). AMPK is a ubiquitously-expressed heterotrimeric enzyme that responds to a rise in the AMP:ATP ratio via activation by upstream kinases such as anti-tumorigenic LKB1 and transforming growth factor β (TGFβ)-activated kinase 1 (TAK1). AMPK can also respond in AMP-independent manner via Ca^+2^/calmodulin-dependent protein kinase (Rubin et al., 2005; Momcilovic et al., 2006; Hardie, 2007; Sanders et al., 2007). Mechanistically, phosphorylation of the catalytic alpha domain at Thr 172 has been reported to be essential for full kinase activity (Woods et al., 2003; Hardie, 2007, 2011). Alternate alpha moieties, such as Ser 173 (Djouder et al., 2010) or 485 (Hurley et al., 2006), have been suggested to serve inhibitory functions on AMPK activity. Functionally, AMPK has been reported to enhance vascular relaxation (Rossoni et al., 2011) and inhibit vessel growth (Nagata et al., 2004). AMPK has also been implicated in *G*_0_/*G*_1_ cytostasis in commercial VSMCs (Igata et al., 2005). These intriguing reports suggesting growth-reducing capacities of AMPK justify further investigation into AMPK as a potential therapeutic target against VSM growth disorders.

Cyclic adenosine monophosphate (cAMP) and its canonical downstream kinase PKA are critical signaling factors that exert a variety of functional effects in cardiac and vascular tissues. It is reported that Thr 197 of the catalytic subunit of PKA is phosphorylated in proportion to PKA activity (Steinberg et al., 1993; Shen et al., 2004) and is a better indicator of activity than the previously reported Ser 338 moiety (Shoji et al., 1983); however, the exact phospho-specific mechanisms of activation of PKA and their impact on kinase efficacy in VSM remain unclear.

It has been previously suggested that a biochemical relationship may exist between AMPK and PKA (Hurley et al., 2006; Djouder et al., 2010; Kim et al., 2010), although the exact nature of this relationship and the extent to which they relate in VSM is uncertain. The cyclic AMP/PKA system, like AMPK, has ability to enhance vasodilation (Zhang et al., 2010; Valero et al., 2011) and inhibit vessel growth (D'Souza et al., 2003; Hewer et al., 2011). Additionally, PKA and AMPK exhibit capacity either discretely or collectively to regulate cellular metabolism, and coordination between their pathways has been reported to exist either synergistically (Cohen and Hardie, 1991; Dyck et al., 1999; Richter et al., 2003) or antagonistically (Watt et al., 2006; Djouder et al., 2010), depending the metabolic demands and the tissue type. Cellular proliferation is normally under tight metabolic control, and although little work has been done to elucidate the relationship between these two signaling systems in the control of cellular growth, crosstalk has been implicated in the inhibition of cancer cell hyperplasia (Han et al., 2009; Lucchi et al., 2011). Therefore, it is intriguing to speculate that crosstalk may exist between PKA and AMPK and that this crosstalk may be of biological significance in its ability to modulate VSM growth.

The purpose of this study was to further characterize the AMPK and PKA signaling pathways and to determine what role(s) they play in the collaborative inhibition of VSM growth. Our hypothesis was that AMPK and PKA possess biochemical crosstalk and, in turn, may act cooperatively to inhibit VSMC proliferation and migration. Novel results show that AMPK controls VSMC proliferation and migration and that these events are regulated at least in part by PKA. These findings offer insight into a potential metabolic signaling network that may provide new therapeutic targets for the reduction of vascular growth disorders.

## Materials and methods

### Materials

AICAR was purchased from Toronto Research Company (North York, ON) and Invitrogen (Carlsbad, CA), Compound C was purchased from Invitrogen, forskolin, and PKI were purchased from Enzo Life Sciences (Farmingdale, NY), and IBMX was purchased from Calbiochem (Darmstadt, Germany). All primary antibodies were purchased from Abcam (Cambridge, MA) or Cell Signaling (Danvers, MA) and were targeted against the following: total AMPK (1:1000), pAMPK_Thr172_ (1:1000), pAMPK_Ser485_ (1:5000), total ACC (1:1000), pACC_Ser80_ (1:1000), total PKA (1:1000), pPKA_Thr197_ (1:1000), total VASP (1:1000), or pVASP_Ser157_ (1:1000), and α-tubulin (1:1000) or beta-actin (1:1000). Antibodies were diluted in IRDye blocking buffer (Rockland; Gilbertsville, PA). For In-Cell Western analysis IRDye anti-rabbit secondary antibodies (1:1000; Rockland) were used for protein detection while Draq 5 (1:10000; Cell Signaling) and Sapphire 7 (1:1000; Li-Cor; Lincoln, NE) were used for DNA staining and protein normalization. Propidium iodide (PI) and RNAse for cell cycle analysis were purchased from Invitrogen.

### Cell culture

This investigation was approved by the Institutional Animal Care and Use Committee and conformed to the Guide for the Care and Use of Laboratory Animals (US National Institutes of Health, Publication No. 85-23, revised 1996). Primary VSMCs were isolated from thoracic aortae of male Sprague-Dawley rats (~125 g; Charles River Labs) by collagenase and elastase digestion and characterized morphologically as previously described (Durante et al., 1993; Joshi et al., 2011). VSMCs were cultured in Dulbecco's Modified Eagles Medium (DMEM) supplemented with Fetal Bovine Serum (FBS, 0.5–10%), 2 mM L-glutamine, and 1:500 dilution of 50 ug/mL Primocin at 37°C in 95% air/5% CO_2_. Cells were serially split and used only through passage 6 to avoid onset of phenotypic switching (Browner et al., 2004). For all assays, *n* is calculated as at least three separate trials each with at least three wells (or reads) per assay from serially split clonal cells.

### Assessment of AMPK and PKA activity

VSMCs were seeded in 96-well plates and, once confluent, were treated with specified pharmacologic agents. After treatment, cells were formalin-fixed, and protein phosphorylation was determined by In-Cell Western analysis as recently described (Joshi et al., 2011; Adderley et al., 2012). Briefly, fixed cells were permeabilized with 0.1% Triton-X, blocked with IRDye blocking buffer and treated with rabbit anti-rat primary antibodies for 1 h at RT. Target proteins were IR-labeled and DNA was stained for protein normalization. Fluorescence was detected and analyzed using Li-Cor Odyssey Infrared Imaging System and software. Additionally, traditional Western blots were performed on primary cell lysates as previously described (Tulis et al., 2000; Mendelev et al., 2009) to verify In-Cell Western data for select experiments. For specific activity, treated cells were lysed in buffer [50 mM Tris, pH 6.8; 1% SDS; 0.1% Triton; protease inhibitor cocktail (Thermo); phosphate inhibitor cocktail (Thermo)], and added to wells of a 96-well microtiter plate. ELISA-based activity assays were performed per manufacturer's instructions. Briefly, for AMPK (MBL International, Woburn, MA), activity was assessed using a mouse insulin receptor substrate phospho-serine 789 reporter with HRP chemiluminescence at 450 nm (Tang et al., 2011; Lin et al., 2012). For PKA (Enzo Life Sciences), activity was measured by tetra-methylbenzidine substrate color development proportional to PKA phosphotransferase activity and measured at 450 nm as described (Cai et al., 2010).

### Phosphatase activity

Cells were harvested in phosphatase inhibitor-free buffer, prepared according to manufacturer's suggestion (Invitrogen), and added to wells of microtiter plates containing 6,8-difluoro-4methyl-umbelliferyl phosphate substrate. When activated by phosphatases, DiFMU was generated in proportion to phosphatase activity, which was read at 450 nm. For specific (PP-2A and PP-2C) phosphatase activities, additional components were added to the buffer per manufacturer's directions that allowed for activity analyses of specific phosphatases within the lysate (Kawaguchi et al., 2001; Pastula et al., 2003).

### Cell cycle analysis

Cells were plated in 12-well plates at 80,000 cells/well in complete media until 50% confluent. Cells were quiesced in 0.5% FBS for 24 h followed by treatment in complete growth media (DMEM, 10% FBS, Primocin) containing select pharmacologic agents for 24 h. As previously stated (Joshi et al., 2012) cells were trypsinized and stained with PI (Invitrogen) per manufacturer's recommendations. The fraction of cells present in each phase of the cell cycle was assessed by flow cytometry (Accuri C6 Flow Cytometer) using CFlow Plus software (Accuri).

### Cell proliferation analysis

VSMCs were plated in 6-well plates at 180,000 cells/well in complete media until 50% confluent. Cells were quiesced in 0.5% FBS for 24 h followed by treatment in complete growth media for 48 h as described above. Cells were trypsinized and proliferation and viability were assessed through automated cell counting with trypan blue exclusion staining (ViCell, Beckman) (Tulis, 2008).

### Cell migration analysis

Following protocols previously described with minor modifications (Liu et al., 2008; Joshi et al., 2011), VSMCs were seeded at 180,000 cells/ml in the upper chamber of a Fluorblok transwell system (BD) in complete media and allowed to adhere. Cells were then treated for 1 h following staining with Cell Tracker Green (10 uM; Invitrogen). Serum-free media was applied to the upper chamber containing the same original treatment and 10 ng PDGF-β was applied to the bottom chamber as a chemoattractant (Yang et al., 2006). Cell migration was assessed from time 0 through 18 h by bottom-read fluorescence at 525 nm (Tecan Infinite M200) with each time point relative fluorescence units (RFU) normalized to time = 0 RFU for each respective condition. Net migration was calculated as a fold change of the total migration for each condition over total control (vehicle) migration at 18 h.

### Statistical analyses

Data were analyzed using Excel 2011 (Microsoft) and Sigma Plot 11.2 (SPSS, Inc.). One-Way analysis of variance (ANOVA) and Tukey's *post-hoc* multiple comparison tests were used to detect changes between individual groups. Two-Way ANOVA with multiple comparisons and Tukey's *post-hoc* tests were used for protein phosphatase (PP) analysis, migration, and cell cycle analysis to detect significance among groups. For each assay, “*n*” is calculated from a minimum of 3 experiments, each consisting of 3 individual wells (and thus, reads) from serially-split, clonal parent cell generations. Data are expressed as mean ± standard error of the mean (SEM), with a *p* < 0.05 considered statistically significant.

## Results

### AMPK enhances PKA activity

**Figure 1:**
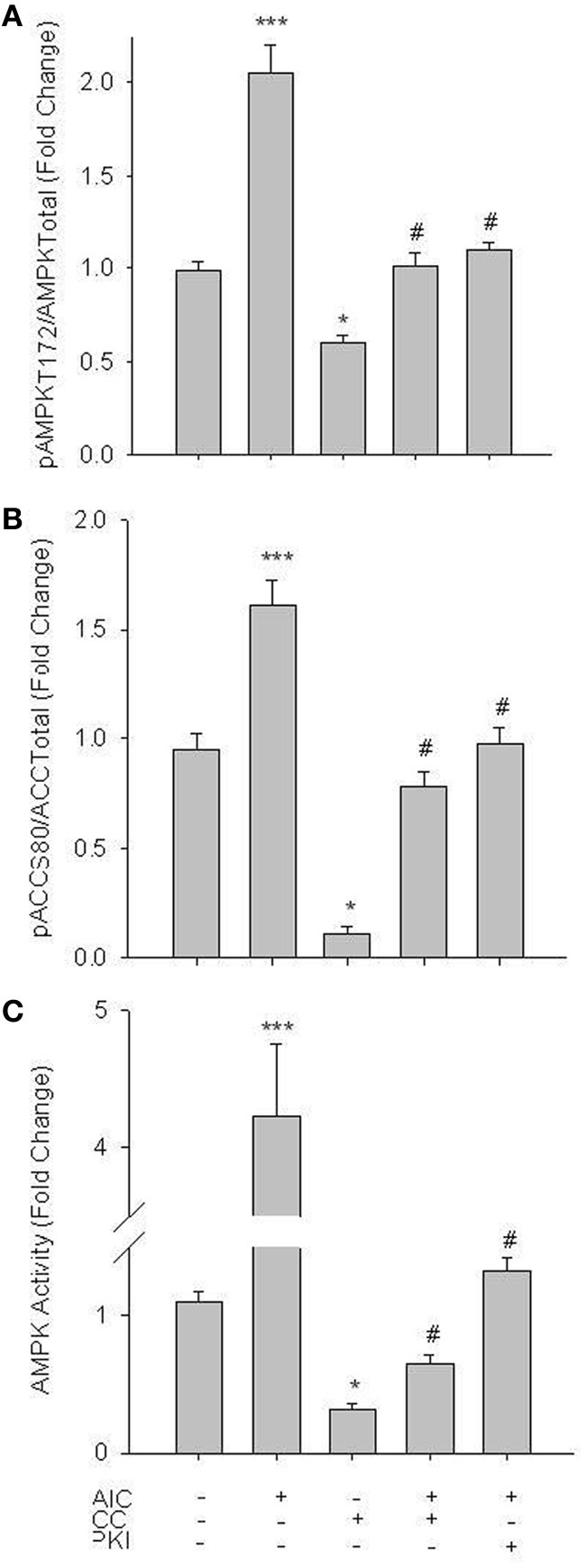
**AICAR increases AMPK signaling in rat primary VSMCs.** Cells were treated with AICAR (1 mM), Compound C (CC; 10 uM) or their combination for 60 min and AMPK Thr172 phosphorylation **(A)**, ACC Ser80 phosphorylation **(B)**, and AMPK activity **(C)** were measured. AICAR significantly increased phosphorylation of both AMPK Thr172 **(A)** and ACC Ser80 **(B)** as well as AMPK activity **(C)**, all in CC-reversible fashion. CC also significantly reduced basal levels of phosphorylated AMPK Thr172, ACC Ser80, and AMPK activity. In all cases addition of the PKA-inhibitor PKI also significantly decreased AMPK signaling and activity. Expression data were normalized to DNA content (Draq 5/Sapphire 700) and presented as phosphorylated AMPK/total AMPK **(A)** or phosphorylated ACC/total ACC **(B)**. Activity was measured via absorbance at 450 nm. *P*-values less than 0.05 were considered statistically significant for *n* = 5–7 per group. ^*^p < 0.05 compared to control; ^***^p < 0.001 compared to control; #*p* < 0.05 compared to respective activator treatment.

Initial experiments focused on activation of AMPK in primary VSMCs. Treatment of cells with 1 mM of the AMPK agonist AICAR (60 min), a concentration previously documented to effectively induce AMPK activity in vascular cells without observable side-effects (Goirand et al., 2007; Chang et al., 2010), significantly enhanced catalytic phosphorylation (Woods et al., 2003) of AMPK Thr172 (Figure 1A) as well as acetyl Co-A carboxylase (ACC) at Ser80 (Figure 1B), a traditional marker of AMPK activity (Witters et al., 1991). Both basal and AICAR-stimulated pAMPK Thr172 and pACC Ser80 were inhibited by 30 min pre-treatment with Compound C (CC; 10 uM), a selective AMPK inhibitor verified for use in VSM (Chang et al., 2010; Song et al., 2011; Sung and Choi, 2011). Intriguingly, inhibition of PKA with PKI (10 uM) also reversed the AICAR-induced increase in both AMPK Thr172 and ACC Ser80 phosphorylation (Figures 1A,B). In agreement, using an AMPK-specific activity assay AICAR induced a significant increase in AMPK activity that was reversed by CC and PKI under stimulated conditions and by CC under basal conditions (Figure 1C).

**Figure 2:**
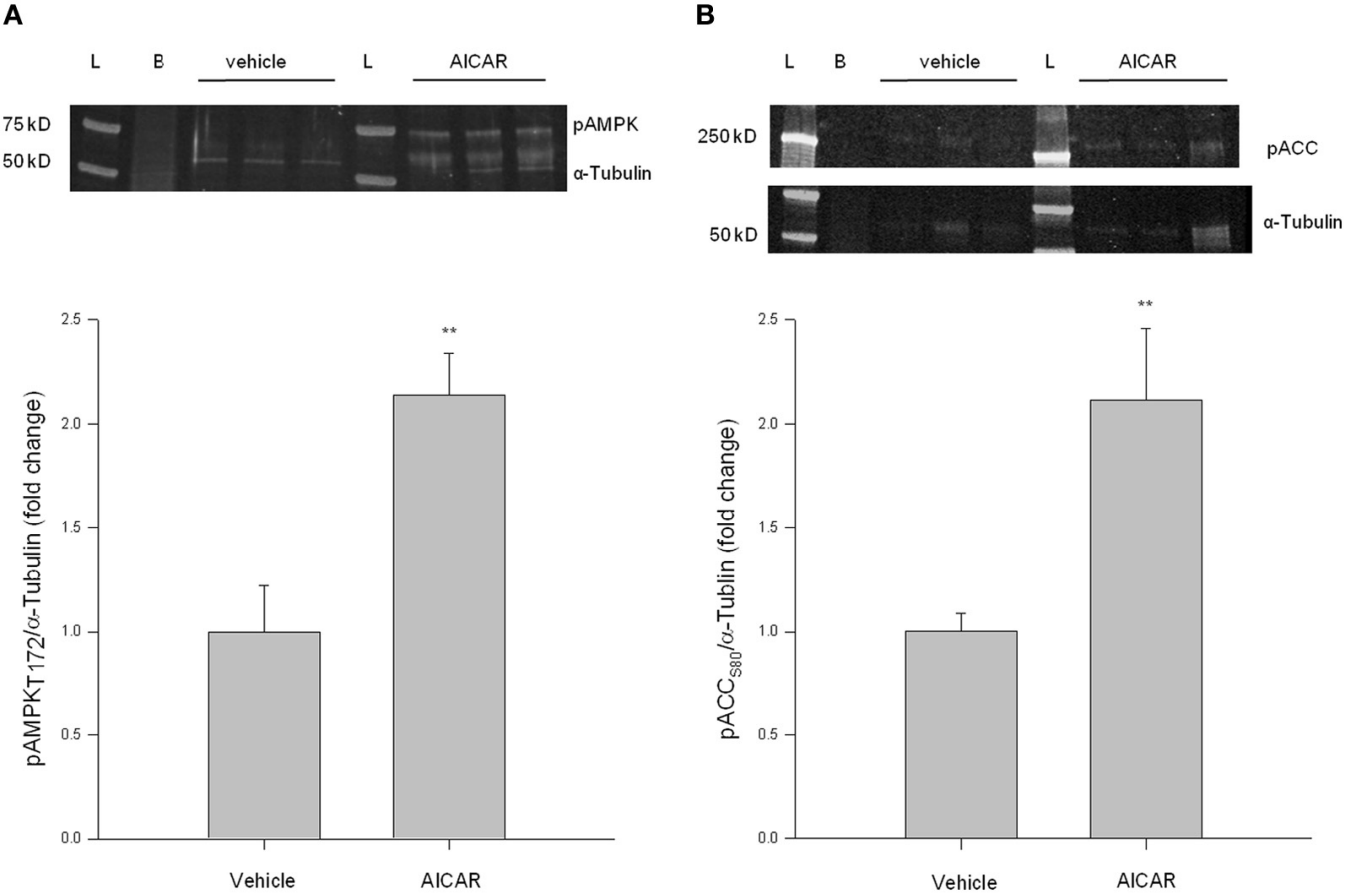
**AICAR increases AMPK signaling in rat primary VSMC lysates.** Cells were treated with AICAR (1 mM) for 60 min and AMPK Thr172 phosphorylation **(A)** and ACC Ser80 phosphorylation **(B)** were measured by traditional Western blotting using IR-linked antibodies. AICAR significantly increased phosphorylation of both AMPK Thr172 **(A)** and ACC Ser80 **(B)**. Phosphorylated protein levels were normalized to α-Tubulin and data presented as fold change over control values. *P*-values less than 0.05 were considered statistically significant for *n* = 3 per group. ^**^p < 0.01 compared to control.

Considering relative novelty of the In-Cell Western approach, traditional Western blotting was used in primary cell lysates to support In-Cell Western results for these initial experiments. VSMC lysates were probed for pAMPK Thr172 and pACC Ser80 as indicators of AMPK activity. As shown in Figure 2, AICAR (1 mM, 60 min) increased AMPK catalytic phosphorylation and downstream ACC signaling (Figures 2A,B). AICAR-mediated increases in pAMPK and pACC were then reduced to control levels in the presence of CC (data not shown). These findings agree with our In-Cell Western results and confirm utility of the In-Cell Western approach in primary VSM preparations.

**Figure 3:**
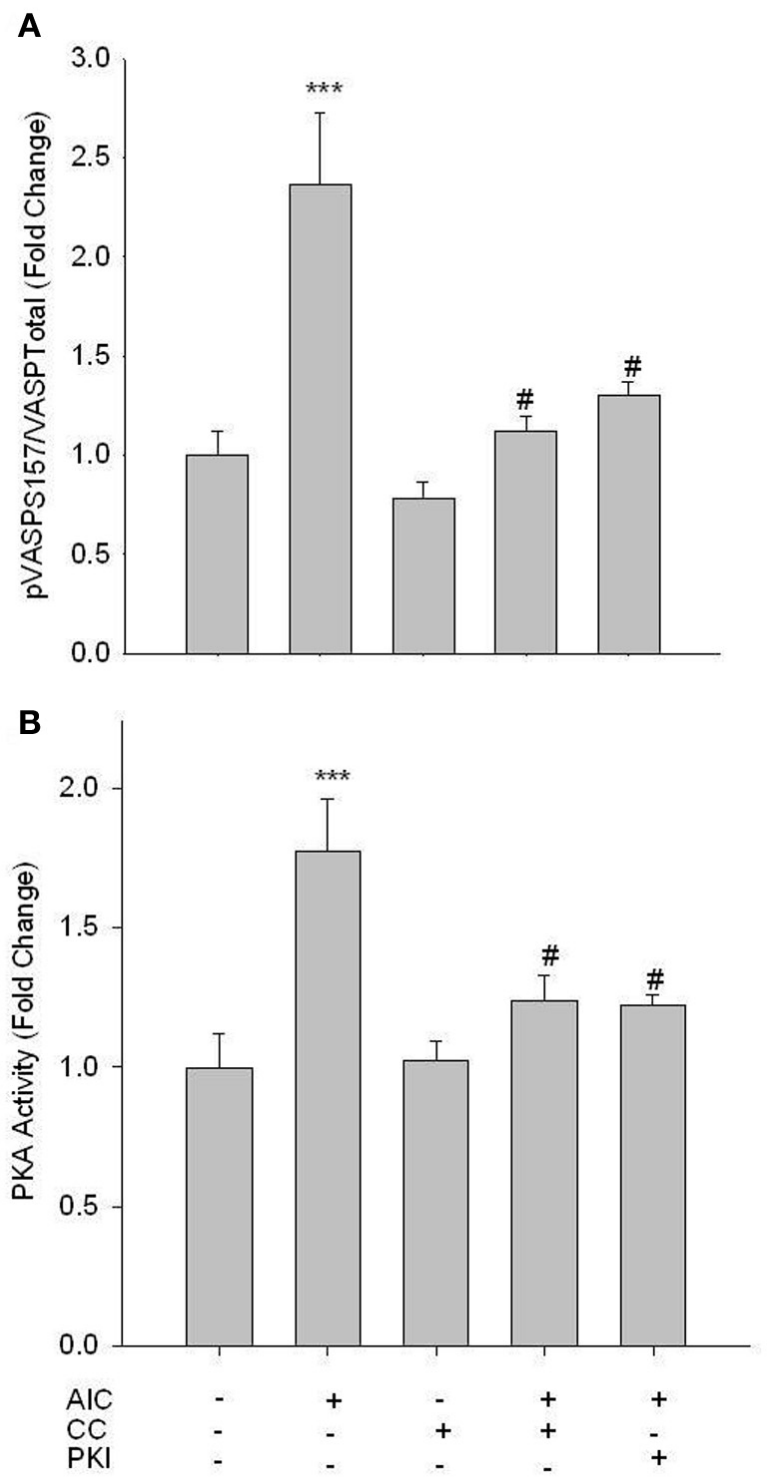
**AMPK enhances PKA activity.** Cells were treated with AICAR (1 mM), Compound C (CC; 10 uM) or a combination of both, and/or PKI (10 uM) for 60 min, and phosphorylation of VASP Ser157 **(A)** and PKA activity **(B)** were measured. In-Cell Western analysis revealed that AICAR significantly increased VASP Ser157 phosphorylation **(A)**. A PKA activity assay revealed that AICAR increased PKA activity **(B)**. Both analyses revealed full reversal with concomitant CC and/or PKI. Data are presented as phosphorylated VASP/total VASP and normalized to DNA content (Draq 5/Sapphire 700) or absorbance at 450 nm for activity. *P*-values less than 0.05 were considered statistically significant for *n* = 5–7 per group. ^***^p < 0.001 compared to control; #*p* < 0.05 compared to respective activator treatment.

Studies were then performed to evaluate the influence of AMPK on PKA signaling. AICAR induced a significant increase in vasodilator-stimulated phosphoprotein (VASP) Ser157 phosphorylation, a reported marker of active PKA (Eckert and Jones, 2007; Joshi et al., 2011), and this was completely reversed by CC (Figure 3A). Additionally, co-treatment of AICAR with a PKA inhibitor, PKI (10 uM; 60 min), also reversed the AICAR-induced phosphorylation of VASP Ser157 (Figure 3A). Furthermore, using an activity assay AICAR induced a significant increase in PKA activity, which was also fully reversed by CC and PKI (Figure 3B). Neither basal levels of pVASP Ser157 nor PKA activity were affected by CC.

### PKA preserves AMPK activity

**Figure 4:**
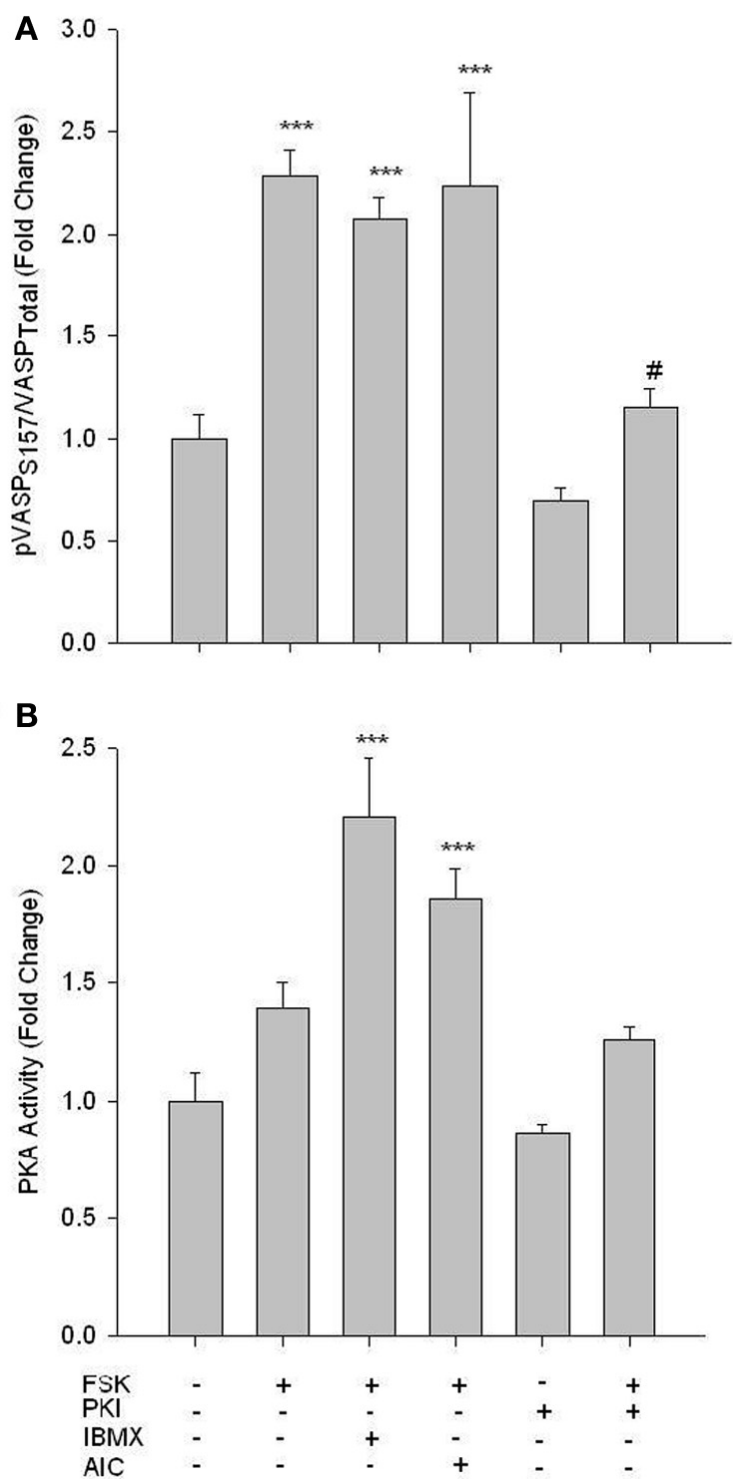
**FSK increases PKA signaling in rat primary VSMCs.** Cells were treated with FSK (10 uM), PKI (10 uM), or FSK and PKI or IBMX (10 uM) or AIC (1 mM) for 60 min, and pVASP Ser157 phosphorylation and PKA activity were determined. In-Cell Westerns revealed that FSK significantly increased pVASP Ser157 alone or in the presence of IBMX and/or AICAR, and these were largely reversed with the PKA inhibitor PKI **(A)**. A PKA activity assay revealed that FSK with IBMX or AIC synergistically increased PKA activity **(B)**. Data are presented as phosphorylated VASP/total VASP and normalized to DNA content (Draq 5/Sapphire 700) or absorbance at 450 nm for activity. *P*-values less than 0.05 were considered statistically significant for *n* = 3–5 per group. ^***^p < 0.001 compared to control; #*p* < 0.05 compared to respective activator treatment.

While phosphorylation of PKA at Thr197 is suggested to be indicative of catalytic activity (Steinberg et al., 1993), neither the adenylyl cyclase agonist Forskolin (FSK, 10 uM; 60 min) nor AICAR induced changes in phosphorylation of this site (data not shown). However, treatment of VSMCs with FSK with or without the broad phosphodiesterase (PDE) inhibitor IBMX (10 uM; 60 min) induced a significant increase in pVASP Ser157, and this was reversed by 10 uM of the PKA inhibitor PKI (Figure 4A). In addition, using an activity assay, while FSK alone induced a non-significant increase in PKA activity, addition of IBMX induced a significant increase in PKA activity that was reversed by PKI (Figure 4B). Additionally, concomitant treatments of FSK and AICAR significantly increased PKA activity (Figure 4B).

**Figure 5:**
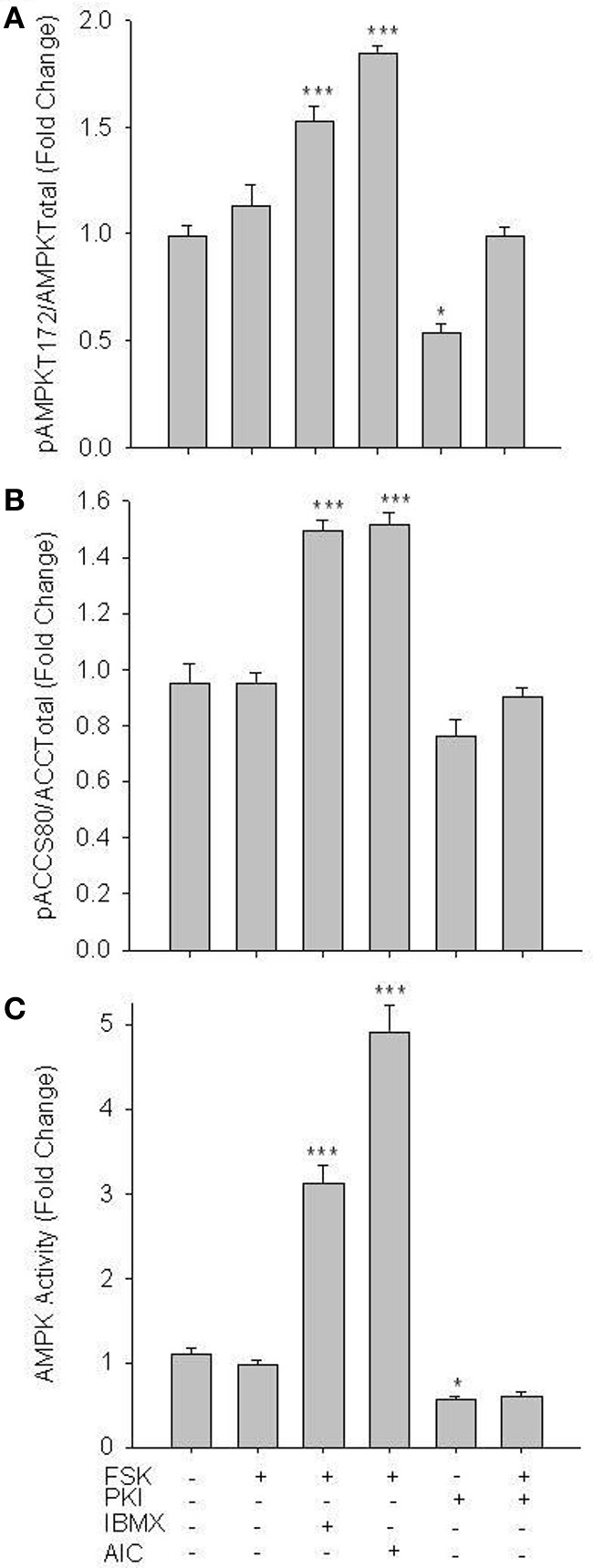
**PKA increases AMPK activity in VSMCs.** Cells were treated with FSK (10 uM), PKI (10 uM), or a combination of FSK and PKI or IBMX (10 uM) or AIC (1 mM) for 60 min. Phosphorylation of AMPK Thr172 and ACC Ser80 were measured by In-Cell Western **(A)** and **(B)** and AMPK activity was measured by pAMPK-specific activity assay **(C)**. FSK alone failed to significantly increase AMPK Thr172 or ACC Ser80 phosphorylation or AMPK activity; however, in the presence of IBMX or AICAR all were significantly elevated. PKI alone significantly reduced both pAMPK Thr172 and activity. Data are presented as phosphorylated AMPK/total AMPK and normalized to DNA content (Draq 5/Sapphire 700) or absorbance at 540 nm for activity. *P*-values less than 0.05 were considered statistically significant for *n* = 5–7 per group. ^*^p < 0.05 compared to control; ^***^p < 0.001 compared to control.

Next, studies were performed to evaluate if PKA mediates changes in AMPK signaling. FSK alone failed to alter pAMPK Thr172; however, when PDE activity was inhibited by IBMX, a significant increase in pAMPK Thr172 was observed (Figure 5A). Intriguingly, synergistic responses in pAMPK Thr172, pACC Ser80, and specific AMPK activity were observed with co-treatments of FSK and AICAR (Figure 5). Also of note, PKI alone induced a significant inhibitory effect on basal AMPK activity, which returned to control levels with FSK co-treatment (Figures 5A,C).

**Figure 6:**
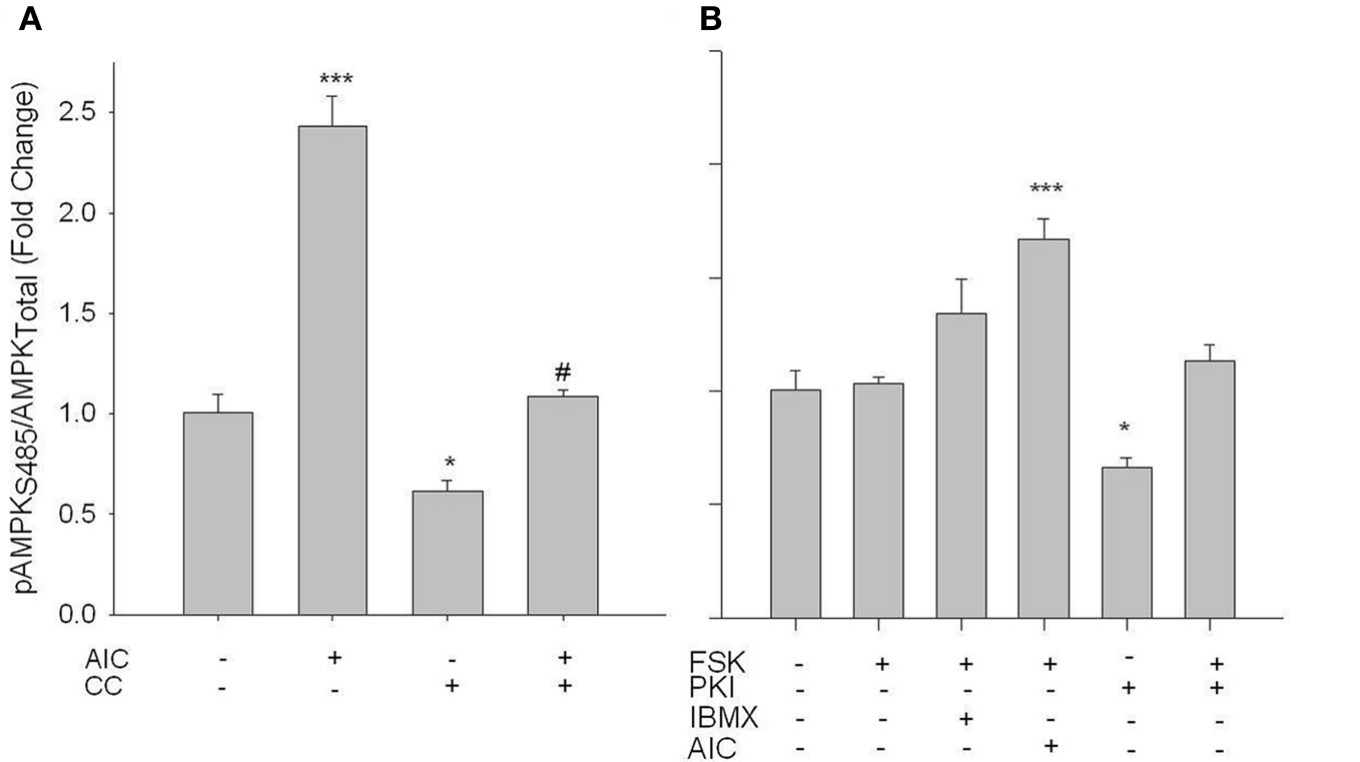
**AICAR increases phosphorylation of AMPK at Ser485 in PKA-independent fashion. (A)** Cells were treated with AICAR (1 mM), Compound C (CC; 10 uM) or a combination of both for 60 min, or **(B)** with FSK (10 uM), PKI (10 uM) or a combination of both, or IBMX (10 uM), or AICAR (1 mM) for 60 min. In-Cell Western analyses revealed that **(A)** pAMPK Ser485 was elevated with AICAR in a CC-reversible fashion. While FSK with/without IBMX had no effect on pAMPK Ser485, FSK + AICAR did significantly increase pAMPK Ser485. Intriguingly, PKI alone significantly decreased Ser485 phosphorylation. Data are presented as phosphorylated AMPK/total AMPK and normalized to DNA content (Draq 5/Sapphire 700). *P*-values less than 0.05 were considered statistically significant for *n* = 5–7 per group. ^*^p < 0.05 compared to control; ^***^p < 0.001 compared to control; #*p* < 0.05 compared to respective activator treatment.

Considering that phosphorylation at Ser485 has been associated with an inhibition of AMPK activity (Stricker et al., 2010; Ning et al., 2011), we investigated pAMPK Ser485 expression under both AMPK- and PKA-stimulated and -inhibited conditions. In primary VSMCs, AICAR induced a significant increase in pAMPK Ser485, which was fully reversed by CC (Figure 6A). CC alone failed to significantly alter basal pAMPK Ser485 levels. While FSK with or without IBMX failed to significantly alter Ser485 phosphorylation, treatment with PKI alone significantly reduced basal pAMPK Ser485, which returned to control levels with concomitant FSK treatment (Figure 6B).

### PKA inhibits protein phosphatases

**Figure 7:**
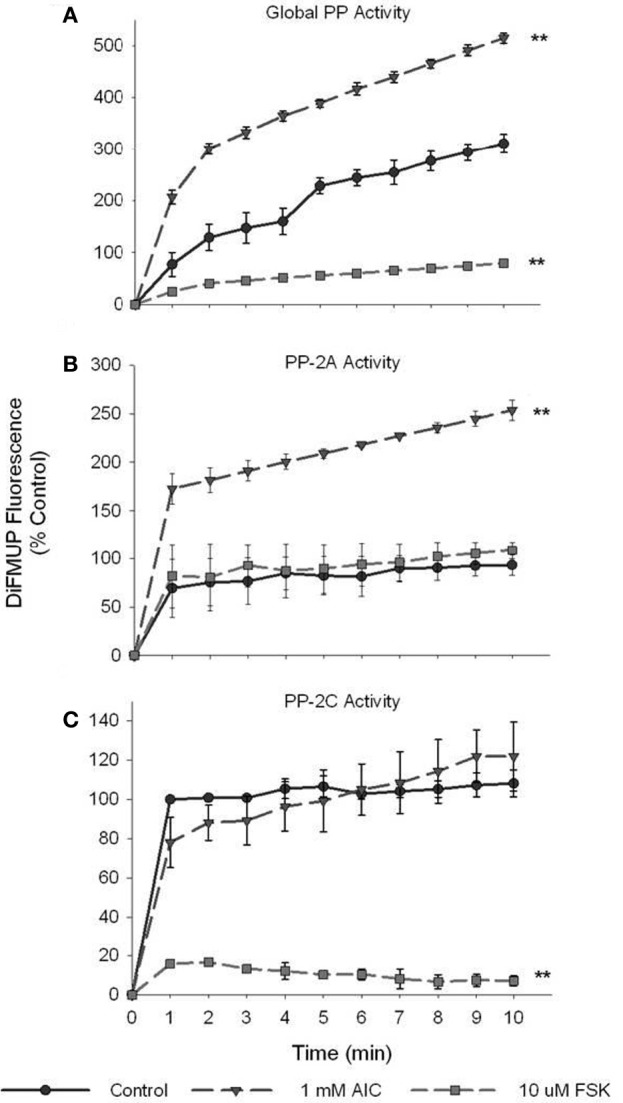
**PKA inhibits global and isoform-specific Ser/Thr phosphatase activity.** Cell lysates were prepared from cells treated with AICAR (1 mM), or a combination of both AICAR and Compound C (CC; 10 uM) for 60 min, or with FSK (10 uM), or a combination of FSK and PKI (10 uM) for 60 min. Phosphatase activity of diluted (1:10) lysate samples was measured by DiFMUP fluorescence at 452 nm after 10 min of incubation in the dark. Activity analysis revealed that AICAR induced while FSK inhibited global Ser/Thr phosphatase activity compared to control values **(A)**. With addition of NiCl_2_ (1 mM) to the reaction buffer, PP-2A-specific activity was tested and revealed that AICAR significantly elevated PP-2A activity **(B)**. Similarly, with addition of MgCl_2_ (20 mM), DTT (2 mM), and EGTA (1 mM) to the assay buffer, PP-2C-specific activity was assessed and revealed that FSK significantly inhibited its activity **(C)**. Two-Way ANOVA with Tukey's *post-hoc* testing was used for multiple comparisons across time points as well as within each treatment group. *P*-values less than 0.05 across time within each group were considered statistically significant for *n* = 3 per group. ^**^p < 0.01 compared to control.

Serine/Threonine PPs, in particular PP-2A and PP-2C (Kemp et al., 2003), have been reported to modulate AMPK activity via kinase dephosphorylation. Therefore, we assessed the ability of PKA to inhibit Ser/Thr PP activity as a possible mechanism of PKA-mediated modulation of AMPK activity. Surprisingly, treatment of VSMCs with AICAR steadily increased global PP activity in significant fashion compared to vehicle-treated control lysates (Figure 7A). Intriguingly, FSK treatment completely inhibited global PP activity in VSMC lysates compared to controls (Figure 7A). With addition of NiCl_2_ (1 mM) to the reaction buffer to specifically monitor PP-2A activity (Pastula et al., 2003), we found that AMPK specifically activated PP-2A in AICAR-treated lysates compared to controls while no difference was detected in FSK-treated cells (Figure 7B). Likewise, when MgCl_2_ (20 mM) was added to the reaction buffer to selectively monitor PP-2C (Pastula et al., 2003), we found that FSK specifically and completely inhibited PP-2C levels compared to controls with no differences detected in AICAR-treated lysates (Figure 7C). Importantly, cumulative reductions in PP activity observed in these experiments (global, PP-2A, PP-2C) were reversible with simultaneous CC- or PKI-inhibition of AMPK or PKA, respectively (data not shown for clarity).

### Cooperative signaling inhibits VSMC proliferation and migration

**Figure 8:**
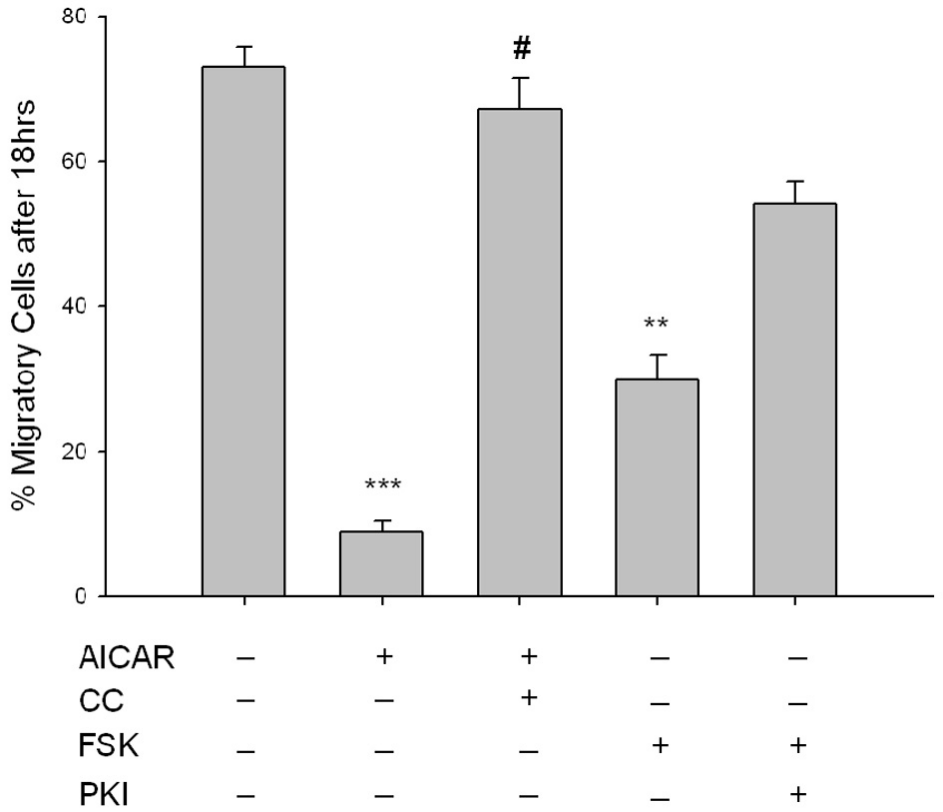
**AMPK inhibits VSMC migration.** Cells were labeled with CellTracker Green and treated throughout the assay with AICAR (1 mM), Compound C (CC; 10 uM) or a combination of both for 60 min, or with FSK (10 uM), PKI (10 uM), or a combination of both for 60 min, and cell migration was determined using a modified Boyden chamber apparatus and bottom-read fluorescence at 525 nm through 18 h. AICAR significantly reduced cell migration across the entirety of the experiment and was reversed by CC. When looking across all time points, FSK had no significant effect on cell migration (*p* = 0.102); however, net migration at time = 18 h was significantly reduced with FSK. The mean score of the best curve fit at 18 h is portrayed by histogram. Significance was determined by Two-Way ANOVA (with Tukey's *post-hoc* testing for multiple comparisons) across the entirety of the 18 h timeframe and at 18 h with all time points compared to vehicle. *P*-values less than 0.05 across time within each group were considered statistically significant for *n* = 5–8 per group. ^**^p < 0.01 compared to control; ^***^p < 0.001 compared to control; #*p* < 0.05 compared to respective activator treatment.

The functional impact of these signaling events was assessed in VSMCs by examination of chemotactic cell migration and cellular proliferation. The influence of AMPK signaling on PDGF-β-stimulated cell migration was assessed using a modified Boyden transwell chemotactic assay over 18 h. AICAR (1 mM) significantly reduced the number of migratory cells which was fully reversed with CC (Figure 8). Interestingly, while only a trend (*p* = 0.102) toward anti-migration was observed with FSK (10 uM) across the entire 18 h experiment, Two-Way ANOVA revealed that the net migration at 18 h was statistically significant compared to controls and was reversed with PKI (Figure 8). Of note, both CC and PKI alone showed no significance across time or at 18 h compared to vehicle controls (data not shown for clarity).

**Figure 9:**
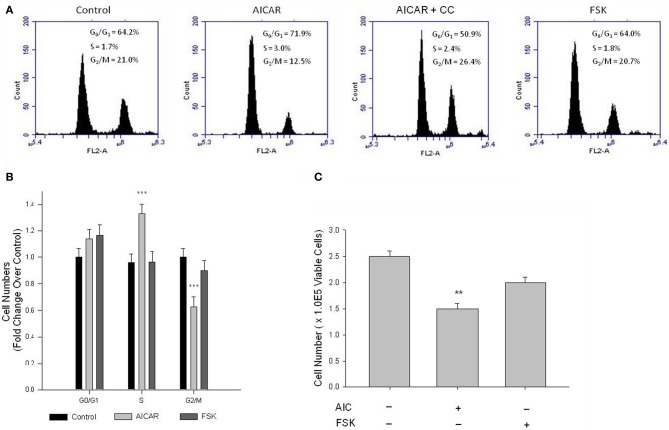
**AMPK induces cell cycle arrest and inhibits VSMC proliferation.** Cells were treated with AICAR (1 mM), with/without Compound C (CC; 10 uM), or with FSK (10 uM) with/without PKI (10 uM) following overnight quiescence. Cell cycle progression was analyzed by flow cytometry using the DNA stain propidium iodide after 24 h **(A)** and cell numbers were quantified after 48 h by automated cell counting and trypan blue exclusion **(B)**. Representative peaks from flow cytometry and histograms of these data illustrate that AICAR significantly inhibits cell cycle progression from S to the G_2_/M phase, revealed by increased numbers in S and reduced numbers in G_2_/M **(A)** and **(B)**, and these were fully reversed by CC **(A)**. Cell cycle analysis revealed that FSK had no effect on cell cycle progression in primary VSMCs **(A)** and **(B)**. Cell count analysis also revealed that AICAR significantly reduced cell numbers after 48 h, and while a trend was observed, Two-Way ANOVA reveals that FSK had no significant effect on cell numbers after 48 h **(C)**. *P*-values less than 0.05 were considered statistically significant after multiple comparisons and Two-Way ANOVA for *n* = 3–5 per group. ^**^p < 0.01 compared to control; ^***^p < 0.001 compared to control (control comparison was made within each stage of the cell cycle for cell cycle analysis).

Cell cycle progression and cell proliferation and viability were examined, respectively, by flow cytometry and automated cell counting with trypan blue exclusion staining. Cell cycle analysis following 24 h serum stimulation revealed AICAR significantly inhibited progression of cells from the S- to G_2_/M-phase, manifested as reduced cell numbers in G_2_/M and elevated cell numbers in S (Figures 9A,B), and these were fully reversed by CC (Figure 9A). FSK did not significantly reduce G_2_/M cell populations or increase G_0_/G_1_-phase cells (Figures 9A,B). Automated cell counts after 48 h serum stimulation revealed that AICAR significantly reduced cell numbers (Figure 9C); however, CC failed to reverse this effect (data not shown). As with the cell cycle data, while a trend was observed FSK alone did not significantly alter cell numbers compared to vehicle controls (Figure 9C).

## Discussion

Findings in this study support our hypothesis that AMPK, in conjunction with PKA, has capacity to inhibit growth of VSM. We show that AMPK inhibits proliferation and migration of rat primary VSMCs, and mechanistic data suggest that these growth-mitigating effects of AMPK are at least partly regulated by PKA. Novel findings also reveal that AMPK can reciprocally modulate PKA activity, suggesting that crosstalk exists between these two pivotal signaling factors. It has been reported in adipocytes and endothelial cells that a biochemical relationship exists between AMPK and PKA (Hurley et al., 2006; Djouder et al., 2010; Kim et al., 2010); however, this relationship has not been established in VSM. Since both cAMP/PKA and AMPK act to regulate cellular metabolism in response to intra- and extra-cellular signals (Cohen and Hardie, 1991; Dyck et al., 1999; Richter et al., 2003; Watt et al., 2006; Djouder et al., 2010), it is reasonable to speculate that a signaling relationship exists between the two signaling cascades in response to energy-consuming cellular processes as proliferation and migration. A synergistic relationship between AMPK and PKA has been suggested in the regulation of cancer growth (Han et al., 2009; Lucchi et al., 2011), and each factor has been implicated individually in VSM growth inhibition (Nagata et al., 2004; Igata et al., 2005; Joshi et al., 2011); therefore, based on the current study we propose a cooperative relationship exists between AMPK and PKA in the control of VSM growth. Data presented here support our theory that AMPK acts to inhibit VSM growth and that PKA acts as an adjuvant to maintain and/or possibly enhance this capacity.

Using rat primary VSMCs, AMPK expression and activity were successfully and specifically induced with AICAR, and this positively influenced the PKA pathway (Figures 1–3). Conversely, the PKA system was successfully induced using FSK, and this led to increased AMPK activity. Following establishment of treatments to specifically modulate AMPK and PKA activity (Figures 1, 4) crosstalk between the two systems was investigated. AICAR increased PKA activity in AMPK-specific fashion (Figure 3), suggesting that AMPK has the ability to increase PKA activity in VSMCs. Paradoxically, as shown in Figure 5 PKA also has the ability to modulate AMPK activity. Although we were unable to detect increases in AMPK Thr172 phosphorylation or AMPK activity from FSK alone, as has been reported in other tissues (Hurley et al., 2006; Mendelev et al., 2009; Djouder et al., 2010; Stricker et al., 2010), under conditions of PDE blockade with IBMX FSK did increase both AMPK Thr172 phosphorylation and activity. This may reflect robust basal PDE activity under cell culture conditions as described recently (Adderley et al., 2012). Additionally, a synergistic effect was observed with concomitant AICAR and FSK treatments, suggesting these two may indeed act cooperatively as biochemical signaling molecules. Moreover, inhibition of PKA by PKI showed a significant reduction in both AMPK phosphorylation and activity, and these were partially restored with concomitant FSK treatment. These findings suggest that PKA may not directly modulate AMPK activity by phosphorylation, but may do so indirectly in a non-Ser172-dependent mechanism, and thus may maintain a basal level of AMPK activity under stimulated conditions.

A suggested mechanism by which PKA may modulate AMPK activity *in vitro* is by inhibitory phosphorylation of AMPK at Ser485 (Hurley et al., 2006; Ning et al., 2011). We tested AMPK Ser485 phosphorylation under stimulatory (10% FBS) and growth-arrested (0.5% FBS) conditions with or without AMPK or PKA stimulation. Our findings reveal that AICAR not only increased catalytic T172 phosphorylation of AMPK but also significantly increased Ser485 phosphorylation, which was completely reversed by CC (Figure 6). Furthermore, PKA inhibition significantly reduced Ser485 phosphorylation, further suggesting a possible regulatory role of PKA on AMPK. Serum-starved cells showed a similar but much lower Ser485 phospho-reduction in response to PKI, but failed to exhibit altered phosphorylation in response to AICAR, CC, and/or FSK treatments (data not shown). Our data suggest that AMPK Ser485 phosphorylation may act to modulate AMPK activity and that PKA may play an important role in regulating this site. In agreement, Hurley et al. showed significant auto-phosphorylation of Ser485 in a cell-free system after just 10 min of incubation with ATP (Hurley et al., 2006). Therefore, expanding on these data we suggest that Ser485 may be a site for auto-phosphorylation and “self-inhibition” of AMPK to fine-tune the dynamic metabolic conditions of stimulated cells on a minute-by-minute basis.

For every kinase, the balance between “on” and “off” is highly regulated and is largely mediated by dephosphorylation by specific PPs. Phosphatase activity, in particularly PP-2A and PP-2C (Kemp et al., 2003), are major contributors to the reduction of AMPK activity. In this light, we examined the ability of PKA to inhibit global and isoform-specific Ser/Thr phosphatase activity. Here we show that PKA stimulation completely inhibits global PP activity and more specifically PP-2C activity in primary VSMC lysates (Figure 7). Taken together with our earlier data showing the PKA inhibition by PKI significantly reduced pAMPK Thr172 and Ser485, a non-kinase mechanism of PKA-mediated regulation of AMPK is likely. Therefore, we suggest that in VSMCs PKA may indirectly mediate AMPK activity via reduced PP-2C activity on AMPK thus inhibiting kinase de-phosphorylation and de-activation. Conversely, specific PP-2A activity was significantly increased in AICAR-treated cells (Figure 7B). While seemingly counterintuitive, we and others (Janssens and Goris, 2001) suggest that PP-2A may play an inhibitory role on cell cycle progression via inactivation of cyclin/CDK complexes providing a possible mechanism by which AMPK acts to inhibit cell cycle progression as our data show (Figure 9). Taken together, these biochemical findings suggest for the first time in primary VSMCs that AMPK and PKA exhibit the capacity for synergistic crosstalk, a finding that may play significant roles in the proposed AMPK-mediated inhibition of VSM growth.

Complementing these biochemical data, functional roles for the cooperative signaling between AMPK and PKA were examined. VSM proliferation and migration are functional mechanisms underlying aberrant vessel growth. Data using a transwell chemotactic assay (Figure 8) clearly show that AICAR significantly inhibits PDGF-β-stimulated VSMC migration. This is the first report demonstrating ability of AMPK to inhibit migration of VSMCs. Additionally, data show that AICAR increases the down-regulatory phosphorylation of VASP at Thr157 (Figure 3A) a protein responsible for actin polymerization, which provides an intriguing hypothetical mechanism for how AMPK disrupts actin dynamics required for migration to occur. Interestingly, after Two-Way ANOVA evaluating time-dependent migration over 18 h FSK had no significant effect on VSMC migration compared to controls; however, overall migration after 18 h showed a significant FSK-mediated anti-migratory effect. Importantly, the degree to which FSK-induced these anti-migratory effects remained significantly higher (29.8 ± 3.6) than the AICAR-mediated effects (8.9 ± 1.5) on VSMC migration. These functional data well-support our biochemical data and together give further credence to our hypothesis that PKA may act to enhance AMPK activity in an indirect manner. In light of these new findings, it is reasonable to postulate that the heretofore PKA-mediated actions within VSMCs that act to modulate proliferation and migration (Joshi et al., 2011) may indeed be AMPK-mediated.

Upon activation, normally contractile VSMCs undergo a phenotypic change and become synthetic and proliferative. Cell cycle analysis after 24 h and automated cell counting after 48 h revealed that AICAR significantly decreased cell cycle progression resulting in a significant reduction in cell numbers (Figure 9). Of note, while AICAR-induced cell cycle inhibition was reversed with CC after 24 h, cell proliferation after 48 h was not affected. A recent study by collaborating investigators revealed that CC alone has capacity to inhibit VSMC proliferation (Mendelev et al., 2009; Peyton et al., 2011), perhaps due to the irreversible inhibitory actions of CC on AMPK (Handa et al., 2011), and in the current study we observed powerful cytostatic effects of CC and AICAR that kept cell numbers at reduced levels. Moreover, in our study while FSK failed to significantly alter cell cycle progression and cell proliferation, a trend toward cytostasis in PKI-reversible fashion was observed (Figure 9). AMPK has been suggested to promote cell senescence; however, these intriguing findings together with our biochemical data suggest that while PKA stimulation alone may not promote cytostasis, PKA may act to enhance AMPK activity thus supporting and/or promoting AMPK-mediated cytostasis. These data provide further evidence for the importance of AMPK in the inhibition of serum-stimulated VSMC growth and shed new light on a signaling network that may provide robust growth reduction in a more specific and targetable manner.

**Figure 10:**
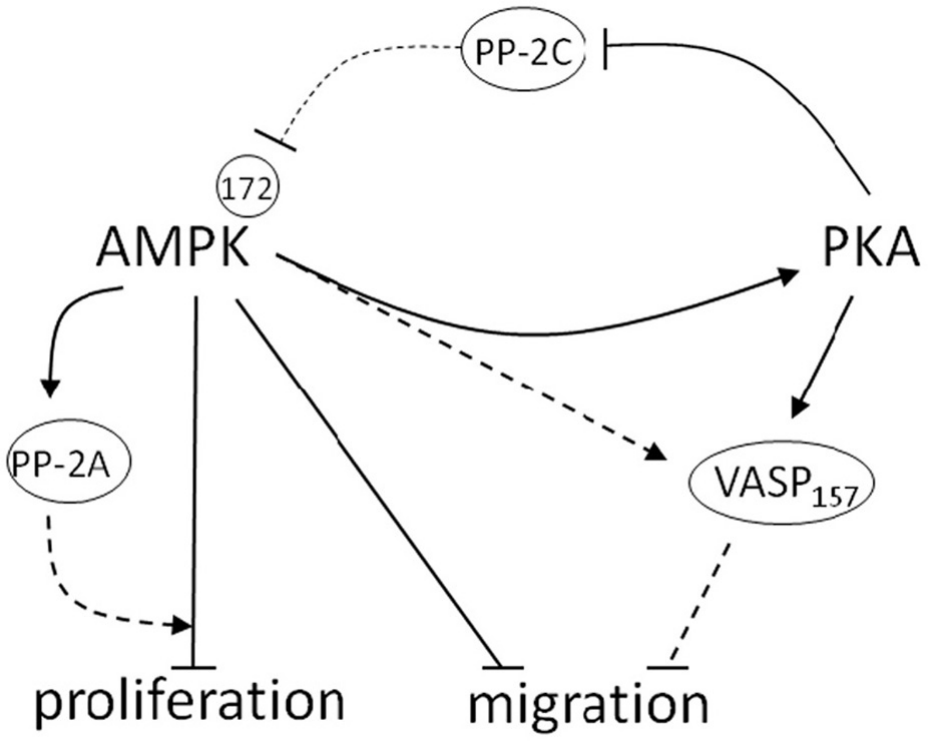
**Schematic depicting the proposed cooperative relationship between AMPK and PKA in the control of VSM growth.** In this diagram blunt lines represent proposed inhibitory while arrows represent proposed stimulatory mechanisms. Those pathways that were not directly tested and remain part of the overall hypothesis of this report are presented as dashed lines. Biochemical data described herein reveal that AMPK increases PKA activity and reciprocally, that PKA may modulate AMPK activity by regulation of proposed AMPK-inhibitory Ser485 phosphorylation. Furthermore, data show that PKA abrogates Ser/Thr phosphatase-2C activity, which may play a crucial role in regulating AMPK activity. Functional data confirm that AMPK reduces VSMC growth by inhibiting cell proliferation and migration. Intriguingly, these data show an increase in reported PKA-specific VASP Ser157 phosphorylation, suggesting AMPK may act directly or indirectly through increased PKA activation to inhibit VASP-directed actin polymerization necessary for cell movement. Additionally, novel findings from this study suggest that AMPK increases PP-2A activity in VSMCs, which provides a possible mechanism for AMPK-mediated cell cycle inhibition, a reported function of PP-2A. Altogether these findings illustrate important cooperative signaling between AMPK and PKA in serum-stimulated VSMCs that may further potentiate the anti-growth properties associated with AMPK.

In conclusion, novel data presented here provide ample evidence that AMPK and PKA communicate biochemically and act in concert as a signaling network capable of controlling growth of VSMCs. This signaling crosstalk may be important in the control of cellular metabolism as has been proposed by others (Cohen and Hardie, 1991; Dyck et al., 1999; Richter et al., 2003; Watt et al., 2006; Djouder et al., 2010), and our data highlight its potential importance in the control of VSM growth. The schematic in Figure 10 highlights the proposed cooperative relationship by which AMPK and PKA are believed to inhibit VSMC proliferation and migration. We suggest that AMPK increases PKA activity and that PKA may independently or in turn inhibit Ser/Thr PP-2C activity, thus indirectly modulating AMPK activity. Additionally, these data suggest that PKA may modulate AMPK activity by regulating phosphorylation of a proposed inhibitory AMPK Ser485 site. These findings suggest a reciprocal and feed-forward mechanism by which PKA modulates AMPK activity in serum-stimulated VSMCs to further potentiate the anti-growth properties associated with AMPK. In addition to its static effects on proliferation, we show for the first time that AMPK has capacity to inhibit VSMC migration, providing a functional mechanism for the prevention of VSMC-mediated vessel remodeling following disease or injury. Intriguingly, our data show an increase in proposed PKA-specific VASP Ser157 phosphorylation, suggesting AMPK may act directly or indirectly through increased PKA activation to inhibit VASP-directed actin polymerization necessary for cell movement. Additionally, novel findings from this study suggest that AMPK increases PP-2A activity in VSMCs, providing a possible mechanism for AMPK-mediated cell cycle inhibition, a reported function of PP-2A. Altogether, these data elucidate a signaling network that has potential clinical importance as a foundation for current therapeutics targeting VSM growth disorders.

## Grants

This project was supported by Award Number R01HL081720 from the National Heart, Lung, and Blood Institute, National Institutes of Health (David A. Tulis) and by an American Heart Association Pre-doctoral Fellowship (Joshua D. Stone). The content is solely the responsibility of the authors and does not necessarily represent the official views of the National Heart, Lung, and Blood Institute, the National Institutes of Health, or the American Heart Association.

### Conflict of interest statement

The authors declare that the research was conducted in the absence of any commercial or financial relationships that could be construed as a potential conflict of interest.
